# Characteristics of Metal Enhanced Evanescent-Wave Microcavities

**DOI:** 10.3390/s100908751

**Published:** 2010-09-21

**Authors:** Takashi Wakamatsu

**Affiliations:** Department of Electrical and Electronic System Engineering, Ibaraki National College of Technology, 866 Nakane, Hitachinaka, Ibaraki, Japan; E-Mail: wakamatu@ee.ibaraki-ct.ac.jp

**Keywords:** resonant microcavity, evanescent wave, optical interface mode, light modulation

## Abstract

This article presents the concept of storing optical energy using a metallic air gap microcavity. Evanescent waves are stored in the air gap of a dielectric/metal/air gap/metal planar microcavity. For an air gap with a micron scale distance between the two metals, incident light excites the optical interface modes on the two metal-air interfaces simultaneously, being accompanied by enhanced evanescent fields. Numerical simulations show that the reflected light depends remarkably upon distributions of the enhanced electric fields in the air-gap at the optical mode excitations. The metallic microcavities have a *Q* value on the order of 10^2^, as determined from calculations. Experimentally, a small mechanical variation of the air-gap distance exhibited a change of reflectivity.

## 1. Introduction

Optical resonators store incident light to form a standing wave mode and are capable of forming large electromagnetic fields internally. The typical Fabry-Perot resonator has a pair of opposing reflection mirrors to generate standing waves with strong fields in an air space or in a transparent dielectric [1,2]. A dielectric multilayer with different refractive indices can also function as an optical resonator, and can generate characteristic standing wave modes internally [2]. The effect of confining light in resonators is a strong light-matter interaction, which enables applications for highly sensitive sensors and high optical-electric conversion devices, *etc.* Furthermore, the standing wave modes play an important role in light emitting devices with a microcavity structure such as organic light-emitting diodes [3–5]. In these optical resonators, an interference of transmitted and reflected light waves in opposing directions to each other is fundamental to optical storage. In contrast with propagating light, an evanescent wave has an imaginary wavenumber part, Im[*k*] and is a localized nearfield standing wave exhibiting exponential decay from an interface [6,7]. The field penetration depth is of the order of wavelength *λ* of the excitation light [6,8]. At the interface of a metal with a large negative dielectric constant, characteristic evanescent waves with enhanced electric fields can be generated by an attenuated total reflection (ATR) method [8–10]. The enhanced fields are utilized as highly sensitive chemical and biological sensors [11,12] because of the high optical energy densities.

Although the general optical cavity storage requires an air-space distance larger than several tens of the wavelength [1,2,7], the evanescent waves of short field-penetration-depth allows the optical storage to have an air gap of wavelength order. Furthermore, the metal evanescent waves enable the optical storage effect to be enhanced due to the formation of strong fields in the narrow air-gap space.

In this article, the author proposes the concept of storing optical energy using a metallic air gap microcavity. We discuss visible evanescent-wave storage in a planar microcavity air gap formed between two opposing metals, and reflection light modulation also which is based on the control of the enhanced evanescent waves in the air gap of submicron order, from wave optics analysis and experimental studies.

## 2. Basic Principle and Experimental Setup

### 2.1. Basic Principle

**Figure 1.**
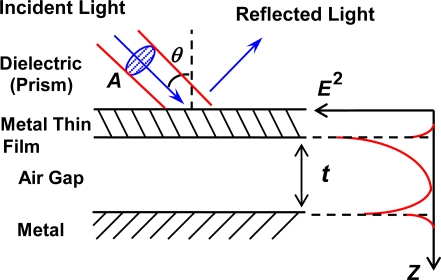
Schematic illustration of the metal enhanced-evanescent-wave microcavity.

The basic principle of metal enhanced-evanescent-wave microcavities - dielectric (prism)/metal thin film/air gap/metal—is shown schematically in Figure 1. The air gap space between the two metals is several times larger than the wavelength *λ* of the incident light.

At a particular incident condition of incident angle *θ* and *λ*, *p*-polarized (TM) incident light through the prism excites the optical interface modes, “ATR modes,” [13] on the two metal/air interfaces simultaneously, in the case of the metals with similar dielectric constants, especially the real part. The ATR mode excitations provide enhancement of the evanescent electromagnetic fields in the air gap of the microcavity, so that the microcavity allows high optical storage.

If the optical resonators store a stationary internal energy, the quality factor (*Q* value) of the resonators, which represents a storage magnitude of optical energy, is given by [1,7]:

(1) $$Q=\frac{\omega \bar{U}}{P_{\mathrm{in}}}$$

where *ω* is the angular frequency of the incident light (period *T* = 2*π* / *ω*), *Ū* is the time-averaged electromagnetic energy in the optical resonators, and *P_in_* is the electromagnetic power incident to the resonators.

We now consider that a plane light wave with a beam cross section *A* is incident to the planar microcavities (multilayer in the *z*-axis direction, see Figure 1). From the time average of the Poynting vector **E** × **H**, *P_in_* is expressed as [7,14]:

(2) $$P_{\mathrm{in}}=\frac{1}{T}\int_{0}^{T}\left|E\times H\right|\mathrm{dt}\cdot A=\frac{1}{2}\frac{{ɛ}_{0}\omega }{k_{0}}E_{0}^{2}A$$

where *ε*_0_ is a permittivity in the vacuum, *k*_0_ is the wavenumber of the incident light, *k*_0_ = 2*π* / *λ*, and *E*_0_ is the magnitude of electric fields of the incident light.

The time-averaged electromagnetic energy in the microcavities is given by:

(3) $$\bar{U}=\frac{1}{T}\int_{0}^{T}\mathrm{dt}\int_{V}\mathrm{dv}\frac{1}{2}(E\cdot D+H\cdot B)=\frac{1}{2}\int_{0}^{\infty }ɛ(z)E{\left(z\right)}^{2}dz\cdot A^{\prime }$$

where *V* in the integral denotes the volume area of the planar microcavities and *A′* denotes the effective cross section inside microcavities. *A′* needs compensation to the cross section *A* of incident light because of oblique incidence of the light ray into the microcavities, *A′* = *A* / cos *θ*.

Hence, we obtain the following expression for the *Q* value for the planar microcavities:

(4) $$Q=\frac{1}{\text{cos}\theta }\left(\frac{2\pi }{\lambda }\right)\int_{0}^{\infty }\frac{ɛ(z)}{{ɛ}_{0}}{\left(\frac{E(z)}{E_{0}}\right)}^{2}\mathrm{dz}$$

The electric fields inside the two metals are considerably weaker than those in the air gap space because of the large negative dielectric constant (real part) of the metals. Therefore, the integral term in Equation (4) is mainly attributed to the enhanced fields in the air gap space, and the *Q* value can be calculated from the distribution of the electric fields in the air gap.

The distributions of enhanced fields between the two metals depend considerably upon the air-gap distance *t*, as demonstrated in later calculation simulations. Since the *Q* value depends upon the *E*-field distributions, as shown in Equation (4), changing the distance in the submicron scale can control the amount of optical energy stored in the air gap space. This effect provides the variation of the *Q* value and performs the modulation of light reflected from the microcavities. Since the largely absorbent metals in the microcavities decrease the *E* fields in the inside, silver metals of small optical absorption were adopted in this study.

### 2.2. Experimental Setup

**Figure 2.**
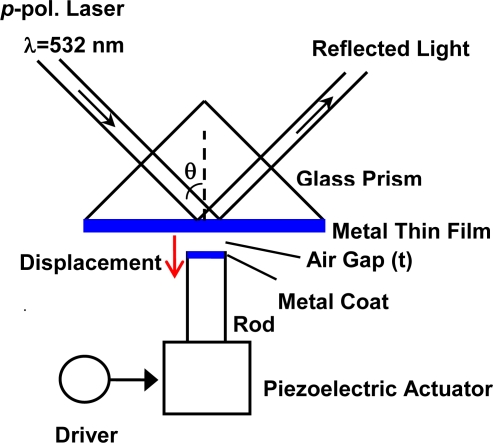
Schematic of the experimental setup for the excitations of enhanced evanescent fields in the microcavity and for successively changing the air-gap distance between two metals.

A light beam derived from a diode pumping solid state (DPSS) laser *λ* = 532 nm was used for demonstration of the evanescent-wave excitations and reflection light modulation, as illustrated in Figure 2. The *p*-polarized light beam of 1-mm diameter was incident to a 45°–right-angle prism of BK-7 glass (*n* = 1.5195 at *λ* = 532 nm) with a thin silver (Ag) film at the ATR-mode excitation angles of 43–45°. The Ag thin film was fabricated on a microscope-cover glass substrate of BK-7 using vacuum evaporation at a thickness of 55 nm; the substrate was fixed on the flat base of the prism with the appropriate immersion oil.

A stainless steel vibrating rod (1.8 mm diameter) was set in the air side at a suitable distance from the Ag thin film. The top of the vibration rod was polished frictionally in plane, to display a mirror face. The flat top was coated with Ag of 1.5-μm thickness using vacuum evaporation. The vibrating rod was attached to the top of a piezoelectric tube. The rough approach of the rod to within piezo range of *t* < 10 μm is performed using an auto-controlled micro-stage. The final positioning for the air gap setting and the rod vibration were achieved by adding an input alternating voltage signal on a DC base signal to the piezo driver. The reflection light passing through the prism was detected using a Si photodiode. The photocurrent AC signals from the optical detector were converted into the voltage forms with a current-voltage amplifier and the output voltage signals were measured using a digital storage oscilloscope.

## 3. Results and Discussion

**Figure 3.**
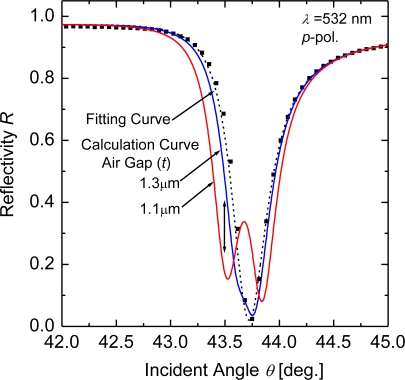
Typical effect of the air-gap distance change upon the reflection from the microcavity of a glass prism/Ag thin film/air gap/Ag. Solid curves represent the calculated reflectivity as a function of incident angle *θ*, for the air-gap distances of *t* = 1.1 and 1.3 μm. The solid quadrangular symbols and the dotted curve respectively represent the measured ATR data at *λ* = 532 nm (*p*-polarized) for a thin Ag film (*d* = 56 nm) and the Fresnel fitting reflectivity.

Figure 3 shows a typical numerical simulation of the air-gap-distance change effect upon reflection at excitations of the ATR modes in the microcavities. The solid curves represent the calculated reflectivity *R* in the ATR configuration with the opposite Ag metal (see Figure 1) for air-gap distances of *t* = 1.1 and 1.3 μm. The *R* curves were calculated using parameters for Ag: the dielectric constant *ε* = −10.9 + i0.37 at *λ* = 532 nm and thickness *d* = 56 nm, which are obtained from the Fresnel reflection-curve-fittings (the dotted curve in Figure 3) for the measured ATR data (solid quadrangular symbols) of the Ag thin film at *λ* = 532 nm. The simulation shows that the existence of the second Ag setting in the air side imparts a change of *R*. The reflectivity change becomes large at around the ATR dip: At the low angle side of *θ* = 43.5°, *R* is varied from 0.474 to 0.146 down with an air-gap distance change of 0.2 μm around 2*λ*, and at the dip angle of *θ* = 43.7°, conversely *R* from 0.089 to 0.337 up. This simulation result indicates that changing the air-gap distance at around the ATR dip provides variation of the optical energy storage in the microcavities, which enables the modulation of the reflection light.

**Figure 4.**
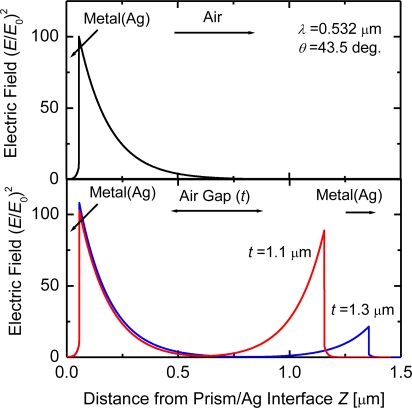
Calculated electric-field-intensity distributions in the thickness direction: the upper figure for the dielectric/metal thin film/air, the lower figure for the dielectric/metal thin film/air gap/metal.

The reflection modulated with the air-gap distance is strongly associated with the distributions of electric fields between the two metals. The electric-field-intensity distributions shown in Figure 4 are calculated as a function of the distance from the prism/metal thin film interface at *θ* = 43.5°; the lower and upper figures depict the structure with and without the opposite Ag metal, respectively. The calculated field intensity is normalized by that of the incident light, (*E*/*E*_0_)^2^. At the incident angle, either electric field is enhanced at the first metal/air interface and decays exponentially. For the structure with the opposite metal, the enhanced fields, however, are excited simultaneously on the second metal also (see the lower figure). The electric field intensities near the second metal interface are increased and the field distributions are modified also when the opposite metal approaches the metal thin film from *t* = 1.3 to 1.1 μm. These distributions of enhanced fields excited on both the metal interfaces depend largely upon the distance between the two metals. The variation of the electric field distributions with changed distance induces the modulation of light reflected from the ATR prism, as shown in Figure 3.

The decay lengths of the enhanced evanescent waves are summarized in Table 1. These values were estimated from the electric field distributions in Figure 4, by curve fittings of an exponential decay function. In the dielectric (prism)-metal-air (the air-gap distance *t* of infinity in Table 1) at *θ* = 43.5°, the enhanced evanescent wave, which is displayed in the upper of Figure 4, has the decay length *d*_1_ of 0.274 μm for *λ* = 0.532 μm. When the enhanced evanescent fields are generated on the second metal/air interface also with decreasing *t*, *d*_1_ is found to become short, from 0.274 down to 0.227 μm. At *t* = 1.1 μm, the two evanescent fields on the metal/air interfaces are of nearly equal decay length. The evanescent waves generated on both the interfaces exhibit a symmetric field distribution, as shown in the lower of Figure 4. Thus the metallic microcavities can store the incident light to form the symmetric enhanced fields inside the air gap.

**Figure 5.**
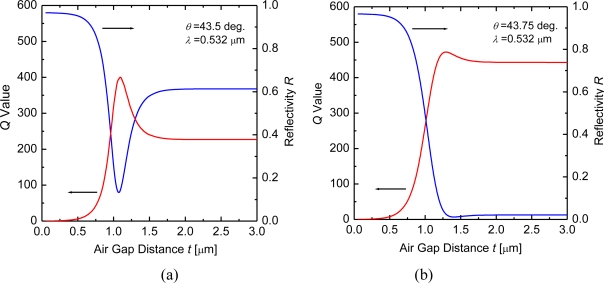
Calculated *Q*-value and reflectivity *R* of the microcavity as a function of air-gap distance *t*: **(a)** at *θ* = 43.5° (the low angle side of the ATR dip); **(b)** at *θ* = 43.75° (the ATR dip angle).

Figure 5 shows the calculated *Q* value and reflectivity *R* as a function of the air-gap distance *t* in the enhanced-evanescent-wave microcavities: Figure 5(a), at *θ* = 43.5°; Figure 5(b), at *θ* = 43.75° (*R* dip angle, see Figure 3). The *Q* values (red curves) and *R* (blue curves) are calculated respectively from the electric-field-intensity distribution (*E*(*z*)/*E*_0_)^2^ in the microcavities by using (4) and the Fresnel formula. The *Q* values of the metal evanescent-wave microcavities are found to be of the order of 10^2^.

The *Q* value curves exhibit the air-gap distance dependence reverse to the *R* curves. The decrease of reflectivity in the microcavities indicates that the incident light energy is absorbed by the metals, which is attributed to the generation of strong evanescent electric fields on the metal interfaces [13]. *Q* value of resonators is generally defined as *ω* times a ratio of stored field energy in the resonators to a loss of energy power [1,7]. From Figure 5, the *Q* value increases unexpectedly, in spite of increasing the absorption loss by the metals. We can interpret that the field enhancement exceeds the increase of the absorption loss. Since another expression for the *Q* value is given by *ω* times a lifetime of photons in the resonators [1,7], this means that the enhanced evanescent fields results in lengthening a lifetime of photons in the metallic microcavities.

In the small air-gap distance of *t* < *λ* (= 0.532 μm), the air gap is not effective on the two parameters of the *Q* value and *R*. The air gap is too narrow to satisfy a total reflection condition on the dielectric and the air. At either incident angles in Figure 5, the narrowness of the air gap disturbs the excitations of the evanescent waves in *t* < *λ* and makes the *Q* value low. The optical responses for the microcavities correspond to a dielectric/metal structure rather than the dielectric/metal/air gap/metal. Therefore, the *Q* values represent approximately zero, because of the high reflectivity and absorption of the metals. On the contrary, in the large air-gap distance of *t* > 3*λ* (= 1.596 μm), the *Q* values become nearly constant for *t* and correspondingly the *R* curves also. This means there is no effect of the second opposing metal departing from the first metal, and the optical behavior is similar to that for the three-layered (dielectric-metal-air) structure.

In the middle air-gap region of *λ* < *t* < 3*λ*, the two parameters largely depend upon the air-gap distance *t*. Naturally, the two parameters have a strong correlation and the dependence of the *Q*-value curves upon *t* is reverse to that of the *R* curves: the *R* and *Q*-value curves have a local minimum and a local maximum, respectively. Comparing Figure 5(a,b), the characteristic for the air-gap distance is largely different from each other and the relationship between the *R* and *Q* value is sensitively changeable for the incident angle *θ*. At the low angle side of *θ* = 43.5° [Figure 5(a)], the *Q* value shows a drastic change for *t* in this air-gap region, and the local maximum of the *Q* value is larger than the *Q* value in the dielectric-metal-air structure (correspondingly, the air-gap region of *t* > 3*λ*) by a factor of 1.77. The *R* value at *θ* = 43.75° [Figure 5(b)] is deeper and the local maximum of the *Q* value is larger. As *t* increased from 0, departing of the second metal from the first metal, the air-gap distance dependence of the two parameters is very similar at either incident angle, but around at *t* beyond the local maximum *Q* values the *R* and *Q*-value curves at *θ* = 43.75° exhibit a slower change. From the respect of a rise in *Q* values as shown in Figure 5, it is therefore found that the optical storage of the microcavities is more effective at the low angle sides than that at the ATR-dip positions.

It is known theoretically and experimentally that enhanced evanescent waves excited on metals by ATR method in the Kretschmann-Raether configuration (prism/metal thin film/air) [8–13] have the maximum fields not at the ATR dip angles where the absorption by the metals becomes the greatest, but at the slightly low angles [10]. This fact can roughly explain the above characteristics of the optical storage effects changing greatly with the small difference of incident angles: at the low angle sides of the ATR dips, the excitations of strong evanescent modes are very changeable for the existence of additional metal approaching to the metal thin films.

The excitation conditions of the enhanced evanescent waves in the microcavities with the air gap, furthermore, are different from that in the Kretschmann-Raether configuration for sole metal thin film. The discrepancy of the excitation conditions can be simply understood from the calculation result that the ATR curve at *t* = 1.1 μm is split into two dips around the reflectivity minimum position as shown in Figure 3.

**Figure 6.**
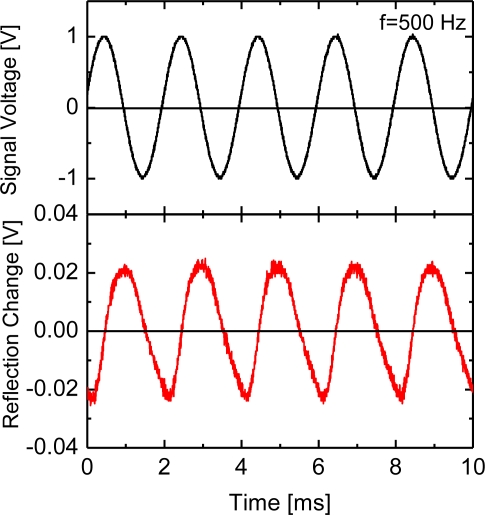
Input signal to the piezo driver for vibration operation at *f* = 500 Hz (the upper figures) and the measured intensity change of light reflected from the microcavity (the lower figures).

The calculations of reflectivity in the enhanced-evanescent-wave microcavities, as shown in Figures 3 and 5, indicate that a variation of the air-gap distance provides modulation of the reflected light. A typical demonstration of the light modulation coupling with the two enhanced evanescent fields is presented in Figure 6.

The upper figure represents an input alternating voltage signal to the piezo driver, which vibrates the Ag-coated rod to control the distance between the two Ag metals; the lower figure shows the intensity change of measured reflection light from the ATR prism. The sine-wave input signal of 1.00 Vp-p at *f* = 500 Hz to the piezo driver (gain × 10) afforded a rod oscillation of 0.12-μm in length.

Although a difference of time phase exists between the two wave signals, the reflection intensity is modulated at the same frequency as that of the corresponding input signal. At a wide frequency range of 20 Hz-2 kHz, a similar modulation of reflection light was also detected and the reflection intensities were varied sensitively with the amplitudes of the input voltage signals. The observed phase difference is probably affected by the mechanical characteristics of the piezoelectric actuator with the metal rod. The magnitude of the reflection modulation at *f* = 500 Hz was 6.8% for *R* = 0.288; its modulation value was less than expected. The reason is probably due to a tilt angle between the vibration rod and the ATR prism base. The top area of the vibration rod set opposite to the optical-mode-excited metal film is valid for light coupling modulation. Tilting of the vibration rod causes the effective coupling area for the excitation light beam on the metal film to be considerably smaller. Increasing the modulation magnitude requires a more precise setting for the tilting.

## 4. Conclusions

The author has described the characteristics of enhanced evanescent waves in the planar metal microcavities with an air-gap distance of light wavelength order. Calculations demonstrate that the electric field distributions of the optical modes simultaneously excited at the two metal interfaces varied remarkably with the air-gap distance, and the *Q* values (10^2^ order) of the microcavities also. Reflection light from the microcavities is strongly associated with the enhanced evanescent fields, and the strong mode-coupling effects enabled the sensitive modulation of the reflection light experimentally.

## Figures and Tables

**Table 1.** Decay lengths of metal evanescent waves.

***t*(μm)**	***d*_1_(μm)**	***d*_2_(μm)**
∞	0.274	–
1.3	0.255	0.255
1.1	0.227	0.255
